# Plasma Carotenoids and Premenstrual Symptoms in a Multi-Ethnic Population of Young Women

**DOI:** 10.3390/nu13113870

**Published:** 2021-10-29

**Authors:** Sophia Kerzner, Tara Zeitoun, Alicia Jarosz, Bibiana Garcia-Bailo, Ahmed El-Sohemy

**Affiliations:** Department of Nutritional Sciences, Temerty Faculty of Medicine, University of Toronto, Toronto, ON M5S 1A8, Canada; sophia.kerzner@medportal.ca (S.K.); tara.zeitoun@mail.utoronto.ca (T.Z.); alicia.jarosz@mail.utoronto.ca (A.J.); bibiana.garcia.bailo@mail.utoronto.ca (B.G.-B.)

**Keywords:** vitamin A, retinol, carotenoids, PMS, premenstrual symptoms

## Abstract

Premenstrual symptoms are experienced by most women of reproductive age, but effective therapies are limited. Carotenoids may have an attenuating effect on premenstrual symptoms; however, studies to date are equivocal. The objective of the present study was to examine the association between plasma concentrations of seven carotenoids and premenstrual symptom severity in 553 women from the Toronto Nutrigenomics and Health study. Participants provided information on fifteen common premenstrual symptoms and severities. Each participant completed a General Health and Lifestyle Questionnaire and provided a fasting blood sample from which plasma carotenoid concentrations were measured. Multinomial logistic regressions were used to determine associations between plasma carotenoid concentrations and premenstrual symptom severity. Beta-cryptoxanthin was associated with moderate/severe increased appetite for women in the highest compared to the lowest tertile (OR: 2.33; 95% CI: 1.39, 3.89). This association remained significant after adjusting for multiple comparisons. There were no observed associations between other plasma carotenoids and any premenstrual symptoms. In summary, higher concentrations of beta-cryptoxanthin were associated with an increased appetite as a premenstrual symptom, but no associations were observed for any other carotenoid and for any other symptom.

## 1. Introduction

Premenstrual Syndrome (PMS) is characterized by a cluster of symptoms that present themselves during the late luteal phase of the menstrual cycle and cease a few days into menses [1]. For women with PMS, these symptoms must be recurring and followed by a symptom-free phase until the end of ovulation. Premenstrual symptoms can include physical, behavioural and psychological effects on women, such as headaches, bloating, fatigue, mood swings, acne, breast pain, anxiety and depression [2,3,4]. Up to 99% of women of reproductive age experience premenstrual symptoms [1,3]. However, considerable variation exists in the type and severity of symptoms that women experience. Some of this variation may be due, in part, to factors such as differences in lifestyle, age, genetics, diet and nutritional status [5,6,7,8,9]. 

Carotenoids are known to contribute to antioxidant defence [10,11], the process by which the body can protect against the effects of reactive oxygen species. Premenstrual symptoms have been associated with an inflammatory process, as evidenced by the positive correlation between their severity and the presence of interleukins and interferon-gamma [12]. Antioxidants are known to have a protective role in reducing inflammation [13], so it is possible that they might protect against certain premenstrual symptoms. 

Vitamin A is an essential fat-soluble vitamin that plays an important role in reproduction through its effects on tissue development, cell differentiation and proliferation [14]. This micronutrient also plays a crucial role in the metabolism of macromolecules, proper vision, brain and immune function [15]. Vitamin A is supplied to the body as either preformed vitamin A or provitamin A, which can be converted into its biologically active form once in the body. Retinol, or preformed vitamin A, supplies approximately 60% of an individual’s average daily vitamin A intake through animal products and by-products such as liver, kidney, eggs, fatty fish and dairy [16,17]. The other 40% is supplied by provitamin A, which includes carotenoids such as beta-carotene, alpha-carotene, and beta-cryptoxanthin [17]. Common dietary sources of provitamin A include dark leafy greens and fruits and vegetables that are red or orange in colour [16,17]. Circulating carotenoids serve as robust biomarkers of fruit and vegetable intake [18].

The potential effects of carotenoids in the amelioration of premenstrual symptoms could involve their antioxidant capacity [11,13]. However, the evidence is limited, and previous studies have yielded inconsistent results [19,20,21,22]. The objective of the present study was to examine the association between plasma concentrations of seven individual carotenoids and premenstrual symptom severity in a multi-ethnic population of young women.

## 2. Materials and Methods

The study participants (*n* = 553) consisted of women of reproductive age from the Toronto Nutrigenomics and Health (TNH) Study. The TNH study is a cross-sectional examination of 1640 young adults aged 20–29 years, living in Toronto and recruited between 2004 and 2010. Women who were breastfeeding or pregnant were excluded from the study. Details of the study have been described previously [23]. Participants completed a General Health and Lifestyle Questionnaire (GHLQ), which included lifestyle, demographic and medical history questions, hormonal contraceptive use and information on physical activity levels (PAL). Fifteen premenstrual symptoms and their respective severities were also self-reported in the GHLQ. The participants recorded their dietary intake via a 196-item Toronto-modified Harvard food frequency questionnaire, had anthropometric measurements taken, and provided an overnight fasting blood sample. The TNH study was approved by the University of Toronto Research Ethics Board. 

Exclusion criteria for the present study included male sex (*n* = 523), those with a reported diagnosis of endometriosis, amenorrhea or polycystic ovarian syndrome (*n* = 14), smokers (*n* = 53), the use of hormonal contraceptives (*n* = 308) and the use of anxiolytics or anti-depressants (*n* = 25), as these medications may affect the severity and/or prevalence of premenstrual symptoms. Individuals with missing GHLQ or plasma carotenoid data (*n* = 164) were also excluded. After all exclusions, the total sample size was 553 women. Participants in this study were categorized into four ethnocultural groups based on self-identification: Caucasian (*n* = 197), East Asian (*n* = 254), South Asian (*n* = 62) or Other (*n* = 40). Caucasians included participants who reported being of European, Middle Eastern, or Hispanic origin. East Asians consisted of individuals of Chinese, Japanese, Korean, Vietnamese, Filipino, Thai or Cambodian origin. South Asians self-reported as Bangladeshi, Indian, Pakistani or Sri Lankan. Participants in the “Other” category included Indigenous Canadians, Afro-Caribbeans and those who were self-reported as belonging to two or more ethnocultural groups.

Participants’ anthropometric variables were measured, including their height, weight, waist circumference and blood pressure, as described previously [3]. Subjects’ body mass index (BMI) was calculated in kg/m^2^. Subjects were also asked to self-report on their PAL in the GHLQ, including time spent sleeping and engaging in light to vigorous physical activity. Physical activity values were converted to metabolic equivalent (MET) hours per week [3]. 

Each participant provided a blood sample following a minimum 12-h overnight fast. Seven plasma carotenoids were measured: beta-carotene, alpha-carotene, retinol, lutein, zeaxanthin, beta-cryptoxanthin and trans-lycopene. Plasma carotenoid concentrations were measured by high-performance liquid chromatography-tandem mass spectrometry as previously described [24]. 

Premenstrual symptom prevalence and severity were self-reported by each female participant in the GHLQ. Women indicated the degree to which they experienced each symptom within 5 days prior to the onset of their period and ending by the 4th day of their period. The questionnaire classified severity as: none, mild, moderate and severe. The questionnaire included the following symptoms: acne, skin blemish; bloating, swelling, breast tenderness; cramping; mood swings, crying easily, irritability, angry outbursts; increased appetite, food cravings; fatigue; headaches; anxiety, tension, nervousness; clumsiness; confusion, difficulty concentrating, forgetfulness; sexual desire/activity change; insomnia; nausea; depression; desire to be alone and other, based on symptoms commonly reported in the literature and previously validated questionnaires [3]. Subjects that selected “other” were asked to specify the symptom; however, due to an insufficient number of responses in this category, these were not included in the analyses. 

All statistical analyses were performed using RStudio (version 1.3.1). The α error was set at 0.05 and all reported *p*-values are two-sided. The distribution of continuous variables was assessed before analysis to evaluate normality; body mass index (BMI) was non-normally distributed and was, therefore, log-transformed for analysis. Premenstrual symptom severity was categorized as none, mild or moderate/severe. The moderate and severe symptom groups were merged into one category for analysis because of the low prevalence of reporting severe symptoms. Plasma carotenoids were categorized into tertiles, with the lowest tertile set as the reference. 

Multinomial logistic regressions were used to determine associations between plasma carotenoid concentrations and the severity of 15 common premenstrual symptoms for beta-carotene, alpha-carotene, retinol, lutein, zeaxanthin, beta-cryptoxanthin and trans-lycopene. *p*-values were calculated for both unadjusted and adjusted models. The adjusted multinomial logistic regressions included the following covariates: age, ethnicity, PAL, medication use, log-transformed BMI and plasma triglycerides. Covariates were selected based on their associations with premenstrual symptoms or plasma carotenoid concentrations in the TNH study population and previous studies. Benjamini-Yekutieli (BY) adjustments for multiple comparisons were applied (α = 0.05: *p* < 0.0083) [25]. Odds ratios and 95% confidence intervals (CI) were reported for all associations. 

## 3. Results

The prevalence of reporting premenstrual symptoms among female TNH Study participants is shown in Table 1. Nearly every woman (99%) in the study population reported experiencing at least one premenstrual symptom, underscoring the high prevalence of this condition among young women during their ovulatory years. Cramps were the most reported premenstrual symptom (76%), followed by mood swings (73%), bloating (71%) and increased appetite (61%). Among these top four reported symptoms, approximately 30% to 40% of women reported experiencing them at a moderate or severe level. Insomnia was the least experienced symptom (12%), with only 3% of women reporting its severity as moderate or higher. 

Multinomial logistic regressions were conducted to examine the associations between plasma carotenoid concentrations and premenstrual symptom severity. Table 2, Table 3, Table 4, Table 5, Table 6, Table 7 and Table 8 show the results for beta-cryptoxanthin (Table 2), beta-carotene (Table 3), alpha-carotene (Table 4), retinol (Table 5), lutein (Table 6), zeaxanthin (Table 7) and trans-lycopene (Table 8). We found several statistically significant associations between specific plasma carotenoids and the severity of premenstrual symptoms after adjusting for age, log-transformed BMI, ethnicity, PAL, use of analgesics and plasma triglyceride levels. However, only a few remained significant after adjusting for multiple comparisons. Women in the highest tertile of plasma beta-cryptoxanthin levels were more likely to experience moderate-severe appetite changes (OR: 2.33; 95% CI: 1.39, 3.89) compared to those in the lowest tertile of beta-cryptoxanthin levels (Table 2). This observation was made both in the unadjusted and adjusted models, (OR: 2.10; 95% CI: 1.29, 3.44) and (OR: 2.33; 95% CI: 1.39, 3.89), respectively. Compared to those in the lowest tertile for plasma beta-carotene, those in the second tertile had a decreased risk of experiencing moderate-severe premenstrual headaches in both the unadjusted and adjusted models, (OR: 0.33; 95% CI: 0.15, 0.73) and (OR: 0.29; 95% CI: 0.13, 0.68), respectively (Table 3). Women in the second tertile of plasma alpha-carotene were less likely to experience mild premenstrual nausea (OR: 0.35; 95% CI: 0.17, 0.73) compared to those in the lowest tertile, although this association was attenuated and no longer significant after adjusting for covariates (Table 4). Women in the second tertile with intermediate retinol plasma concentrations were more likely to experience moderate/severe premenstrual sexual desire changes compared to those in the lowest tertile (Table 5). This association was observed in both unadjusted and adjusted models (OR: 2.35; 95% CI: 1.31, 4.23) and (OR: 2.17; 95% CI: 1.17, 4.01), respectively. There was an inverse association between high plasma lutein concentrations and moderate/severe premenstrual cramps (OR: 0.48; 95% CI: 0.29, 0.81); however, this association was attenuated after adjustment for covariates (Table 6). Women in the second tertile of plasma lutein levels had lower odds of experiencing mild premenstrual mood swings in comparison to those with low plasma lutein levels (OR: 0.48; 95% CI: 0.28, 0.83). This association was observed after adjusting for potential covariates only. There were no observed associations between plasma trans-lycopene or plasma zeaxanthin with any premenstrual symptom severities (Table 7 and Table 8). 

## 4. Discussion

The present cross-sectional study of 553 young women living in Canada examined the association between plasma carotenoids and retinol on the severity of 15 common premenstrual symptoms. While previous research on this topic assessed retinol alone [19,20,21,22], the current study included six different plasma carotenoids along with retinol to provide a more comprehensive understanding of the role of vitamin A and provitamin A carotenoids, which may have unique biological effects. Further, using serum concentrations as opposed to dietary intake allowed a more direct measure of exposure as serum values take into account the variability of an individual’s metabolism. We observed a positive association between plasma beta-cryptoxanthin concentrations and increased appetite/food cravings during the premenstrual period, which remained significant after adjusting for potential confounders and accounting for multiple comparisons. No other plasma carotenoids or retinol were associated with the severity of the 15 common premenstrual symptoms examined in the present study after adjustments for multiple comparisons.

Provitamin A in the form of carotenoids, the brightly coloured pigments in most orange and yellow foods, is common in the diet and acts as a powerful antioxidant when in carotenoid form. Recent studies have shown that beta-cryptoxanthin, commonly found in squash, hot chilli peppers and tangerines, are absorbed more readily than other carotenoids, giving it a bioavailability from carotenoid-rich foods that is considerably higher than beta-carotene, which is traditionally considered to have the highest biological conversion activity and is used as a marker of provitamin A physiological status [14,26]. Once digested, beta-cryptoxanthin is absorbed through facilitated transport at low physiological concentrations [26]. Passive diffusion supplements this process with higher pharmacological doses. It then undergoes a biosynthetic conversion to retinol in the enterocytes; however, this conversion process is poorly understood [26]. Other common carotenoids include alpha-carotene, zeaxanthin, and lutein. Vitamin A forming carotenoids, such as beta-carotene and beta-cryptoxanthin, are converted into retinol once in the body. However, due to genetic variability, humans have varying abilities to carry out this conversion process, and therefore, retinol is the most reliable marker of vitamin A [27]. While carotenoids function and have biological effects as precursors for vitamin A, they also function as antioxidants and, through these effects, may play a role in premenstrual symptoms [11]. 

Previous research suggests that antioxidants may decrease the incidence of certain chronic diseases because of their role in reducing oxidative damage and inflammation [13]. Carotenoids are amongst the strongest antioxidants, suggesting a potential ameliorating effect on premenstrual symptoms through this pathway [11]. Studies have shown that inflammatory markers, such as the interleukins 2, 4, 10 and 12, along with interferon-gamma, are positively associated with premenstrual symptom severity [12]. Elevated serum concentrations of high-sensitivity C-reactive protein (hs-CRP), a molecule elevated during conditions of inflammation, were associated with a score of menstrual symptoms, where symptoms were categorized into one of four groups: behaviour, mood, pain or physical symptoms [28]. Another cross-sectional study reproduced these results, showing a significant positive association between hs-CRP concentrations and the following premenstrual symptoms: mood swings, abdominal cramps/back pain, appetite cravings/weight gain/bloating and breast pain [29]. These results suggest that anti-inflammatory agents, such as antioxidants, may play a role in the prevention or treatment of some premenstrual symptoms. 

Given the limited number of studies on carotenoids and premenstrual symptoms [19,20,21,22], the present study examined whether plasma carotenoid concentrations were associated with premenstrual symptoms in young women of reproductive age. Based on their role as antioxidants, it was hypothesized that carotenoids would be associated with a lower risk of premenstrual symptoms; however, circulating concentrations of several common carotenoids were not associated with any symptoms. Furthermore, beta-cryptoxanthin showed a positive association with an increased appetite and food cravings. This finding may be due, in part, to differences in the biological activity of different carotenoids. Carotenoids can be classified into two main categories: vitamin A forming carotenoids and non-vitamin A forming carotenoids [26]. Lycopene is an example of a non-vitamin A forming carotenoid, while beta-cryptoxanthin is an example of a vitamin A forming carotenoid and is converted to vitamin A once in the body. It is possible that our observations of increased severity of food cravings within the highest tertile of beta-cryptoxanthin is due to reverse causation, where women who experience moderate/severe food cravings during their menses increase their dietary consumption to meet their new hunger requirements, and in doing so, may transiently increase their intake of beta-cryptoxanthin containing foods. 

Results from previous studies on vitamin A and premenstrual symptoms have been inconsistent. Frankel and colleagues recently evaluated the relationship between serum antioxidant vitamins, such as vitamins A, C and E, and PMS in 259 healthy women aged 18–49 in western New York, U.S.A [21]. There were no associations between serum vitamin A, measured using retinol as a biomarker, and the prevalence or the severity of PMS, a result that is consistent with the findings of the present study. Bahrami and colleagues also recently investigated the associations between serum fat-soluble vitamin concentrations, such as vitamin A and E, and PMS in 897 Iranian adolescents and found that serum vitamin A, measured by trans retinol, was inversely associated with the presence of PMS [22]. The discrepancy in results may potentially be due to the ethnic differences in the occurrence and severity of premenstrual symptoms, where a few observational studies have noted a higher prevalence of premenstrual symptoms in certain ethnic groups [3,30]. 

While most of the research on this topic assesses retinol alone as a marker for vitamin A, one strength of the present study was that it compared seven different plasma carotenoids to provide a more comprehensive understanding of the role of vitamin A and the individual carotenoids. Further, using serum concentrations, as opposed to dietary intake, allowed a more accurate measure of exposure, as serum values take into account the variability of an individual’s metabolism. The large amount of data collected in the present study population enabled us to adjust for a number of factors known to influence premenstrual symptoms and carotenoid levels, thereby reducing the likelihood of residual confounding. Lastly, this study represents a multi-ethnic population, whereas most other studies have focused on homogeneous groups of women.

The present study is not without its limitations. A retrospective questionnaire was used to report premenstrual symptom severities, which could result in over or underestimation of the prevalence and severities of some symptoms. The participants were also young adults recruited from a large urban university campus, so the results may not be representative of all menstruating females. Although the timing of blood sample collection for carotenoids was not particular to the luteal phase, it is not clear whether beta-cryptoxanthin levels differ during the menstrual cycle. A previous study assessed serum carotenoid variations across the menstrual cycle but did not measure beta-cryptoxanthin [31]. Findings from that study show that serum retinol was the only carotenoid associated with higher estradiol and testosterone during menses, while beta-carotene, lycopene and lutein were not associated with any reproductive hormones related to the menstrual cycle.

The majority of women who menstruate experience painful and undesirable premenstrual symptoms that last up to 9 days a month. The average age range between menarche and menopause is from 12 to 51 years. This translates into almost 500 cycles in a woman’s lifetime. In the present study population, 99% of women reported experiencing at least one premenstrual symptom (Table 1). While hormone-based medications can significantly decrease symptom discomfort for many women, these medications can be expensive, can be accompanied by various undesirable side effects and have been shown to affect biomarkers of inflammation [32]. Identifying dietary strategies to manage premenstrual symptoms may provide an easily implementable, economical alternative to the cost and potential side effects of hormonal contraceptives.

## 5. Conclusions

The findings from the present study suggest that higher plasma beta-cryptoxanthin concentrations may be associated with an increase in premenstrual appetite and food cravings. This finding, however, might be due to reverse causation, with an increased premenstrual appetite leading to increased consumption of foods high in beta-cryptoxanthin. No other carotenoids were associated with differences in severity of any premenstrual symptoms after adjusting for multiple comparisons. Further research of the relationship between dietary factors and individual premenstrual symptoms may yield a better understanding of the role of diet in premenstrual symptom prevalence and severity and potentially lead to the development of dietary strategies to manage these symptoms. 

## Figures and Tables

**Table 1:** Prevalence of reporting of premenstrual symptoms among 553 women.

Premenstrual Symptoms	Prevalence by Degree of Reported Severity (*n* (%))			Total Presence of Symptoms
	None	Mild	Moderate/Severe	
Any symptom				549 (99)
Cramps	130 (23)	187 (34)	236 (43)	423 (76)
Mood swings/crying easily/irritability/angry outbursts	152 (27)	208 (38)	193 (35)	401 (73)
Bloating/swelling/breast tenderness	158(29)	213 (38)	182 (33)	395 (71)
Increased appetite/food Cravings	212 (38)	160 (29)	181 (33)	341 (61)
Acne/skin blemish	226 (41)	214 (39)	113 (20)	327 (59)
Fatigue	247 (45)	176 (32)	130 (23)	306 (55)
Sexual desire/activity change	283 (51)	177 (32)	93 (17)	270 (49)
Desire to be alone	351 (63)	136 (25)	62 (12)	202 (37)
Anxiety/tension/nervousness	352 (64)	129 (23)	77 (13)	201 (36)
Depression	398 (72)	98 (18)	57 (10)	155 (28)
Headache	412 (75)	90 (16)	51 (9)	141 (24)
Confusion/difficulty concentrating/forgetfulness	423 (76)	87 (16)	43 (8)	130 (23)
Nausea	464 (84)	68 (12)	21 (4)	89 (16)
Clumsiness	470 (85)	59 (10)	25 (5)	83 (15)
Insomnia	485 (88)	52 (9)	16 (3)	68 (12)

**Table 2:** Unadjusted and adjusted Odds Ratios and 95% CIs for the association between tertiles of plasma beta-cryptoxanthin and premenstrual symptoms.

Symptom	Tertiles ^1^	Mild vs. None				Moderate/Severe vs. None			
		OR (95% CI) ^2^	*p* ^2^	OR (95% CI) ^3^	*p* ^3^	OR (95% CI) ^2^	*p* ^2^	OR (95% CI) ^3^	*p* ^3^
Acne/skin blemish	T1	1	1	1	1	1	1	1	1
	T2	1.47 (0.92, 2.33)	0.1	1.51 (0.94, 2.43)	0.08	1.26 (0.72, 2.20)	0.42	1.33 (0.75, 2.37)	0.32
	T3	1.14 (0.72, 1.81)	0.58	1.13 (0.70, 1.83)	0.62	1.14 (0.66, 1.97)	0.65	1.26 (0.71, 2.27)	0.43
Desire to be alone	T1	1	1	1	1	1	1	1	1
	T2	0.64 (0.39, 1.06)	0.08	0.64 (0.39, 1.06)	0.08	0.58 (0.30, 1.11)	0.1	0.60 (0.31, 1.16)	0.13
	T3	0.76 (0.46, 1.24)	0.27	0.72 (0.44, 1.21)	0.22	0.91 (0.50, 1.66)	0.76	1.10 (0.57, 2.02)	0.83
Anxiety/tension/nervousness	T1	1	1	1	1	1	1	1	1
	T2	1.22 (0.75, 1.97)	0.42	1.19 (0.73, 1.93)	0.49	0.55 (0.27, 1.14)	0.11	0.57 (0.28, 1.19)	0.13
	T3	1.01 (0.62, 1.67)	0.96	0.90 (0.53, 1.50)	0.67	1.24 (0.68, 2.27)	0.48	1.35 (0.71, 2.56)	0.35
Increased appetite/food cravings	T1	1	1	1	1	1	1	1	1
	T2	1.04 (0.64, 1.69)	0.88	1.07 (0.65, 1.76)	0.78	1.18 (0.72, 1.96)	0.51	1.23 (0.74, 2.05)	0.42
	T3	1.09 (0.66, 1.83)	0.73	1.26 (0.74, 2.15)	0.4	2.10 (1.29, 3.44)	0.003	2.33 (1.39, 3.89)	0.001
Bloating/swelling/breast tenderness	T1	1	1	1	1	1	1	1	1
	T2	0.65 (0.39, 1.09)	0.1	0.66 (0.40, 1.11)	0.11	0.66 (0.40, 1.12)	0.12	0.70 (0.41, 1.18)	0.41
	T3	1.06 (0.63, 1.79)	0.81	1.10 (0.65, 1.89)	0.71	0.91 (0.53, 1.56)	0.74	1.08 (0.62, 1.89)	0.62
Clumsiness	T1	1	1	1	1	1	1	1	1
	T2	1.93 (1.00, 3.70)	0.04	1.84 (0.95, 3.57)	0.07	0.63 (0.18, 2.19)	0.47	0.67 (0.19, 2.44)	0.55
	T3	1.57 (0.80, 3.09)	0.19	1.42 (0.70, 2.84)	0.33	1.41 (0.51, 3.87)	0.51	1.66 (0.54, 5.09)	0.37
Confusion/difficulty concentrating/forgetfulness	T1	1	1	1	1	1	1	1	1
	T2	0.89 (0.49, 1.61)	0.7	0.81 (0.44, 1.49)	0.5	0.79 (0.34, 1.79)	0.56	0.81 (0.35, 1.87)	0.63
	T3	1.49 (0.86, 2.58)	0.16	1.23 (0.70, 2.19)	0.47	1.20 (0.55, 2.57)	0.65	1.27 (0.56, 2.87)	0.56
Cramps	T1	1	1	1	1	1	1	1	1
	T2	0.81 (0.46, 1.42)	0.45	0.85 (0.48, 1.51)	0.58	0.78 (0.45, 1.33)	0.36	0.86 (0.50, 1.51)	0.61
	T3	0.72 (0.41, 1.26)	0.25	0.84 (0.47, 1.50)	0.56	0.66 (0.39, 1.13)	0.13	0.88 (0.51, 1.56)	0.68
Depression	T1	1	1	1	1	1	1	1	1
	T2	0.89 (0.51, 1.54)	0.67	0.86 (0.49, 1.50)	0.59	0.68 (0.33, 1.41)	0.3	0.69 (0.33, 1.43)	0.32
	T3	1.05 (0.61, 1.80)	0.87	1.08 (0.61, 1.91)	0.79	1.10 (0.57, 2.13)	0.77	1.17 (0.59, 2.33)	0.65
Fatigue	T1	1	1	1	1	1	1	1	1
	T2	1.16 (0.72, 1.87)	0.53	1.17 (0.72, 1.90)	0.52	1.18 (0.70, 2.00)	0.54	1.19 (0.70, 2.02)	0.53
	T3	1.45 (0.90, 2.33)	0.13	1.45 (0.88, 2.38)	0.14	1.45 (0.86, 2.46)	0.16	1.37 (0.80, 2.38)	0.25
Headaches	T1	1	1	1	1	1	1	1	1
	T2	1.19 (0.70, 2.07)	0.53	1.30 (0.74, 2.29)	0.36	1.21 (0.60, 2.44)	0.58	1.25 (0.61, 2.58)	0.54
	T3	0.85 (0.48, 1.51)	0.58	0.93 (0.51, 1.71)	0.81	0.75 (0.35, 1.60)	0.45	0.92 (0.41, 2.04)	0.83
Insomnia	T1	1	1	1	1	1	1	1	1
	T2	0.83 (0.42, 1.64)	0.59	0.89 (0.44, 1.79)	0.75	0.22 (0.05, 1.02)	0.05	0.24 (0.05, 1.13)	0.07
	T3	0.73 (0.36, 1.48)	0.38	0.68 (0.32, 1.45)	0.32	0.54 (0.18, 1.65)	0.18	0.63 (0.19, 2.07)	0.45
Mood swings/crying easily/irritability/angry outbursts	T1	1	1	1	1	1	1	1	1
	T2	1.19 (0.71, 1.99)	0.5	1.24 (0.73, 2.09)	0.42	1.06 (0.62, 1.79)	0.84	1.11 (0.65, 1.89)	0.7
	T3	0.90 (0.54, 1.52)	0.71	0.82 (0.48, 1.41)	0.48	1.14 (0.68, 1.90)	0.62	1.22 (0.71, 2.09)	0.47
Nausea	T1	1	1	1	1	1	1	1	1
	T2	1.87 (0.97, 3.62)	0.06	1.96 (0.99, 3.84)	0.05	0.98 (0.37, 2.63)	0.98	1.25 (0.45, 3.47)	0.67
	T3	0.95 (0.46, 2.00)	0.9	1.17 (0.54, 2.55)	0.68	0.91 (0.34, 2.41)	0.84	1.58 (0.53, 4.67)	0.41
Sexual desire/activity change	T1	1	1	1	1	1	1	1	1
	T2	0.96 (0.45, 1.15)	0.87	0.99 (0.62, 1.58)	0.96	0.58 (0.33, 1.03)	0.06	0.61 (0.34, 1.11)	0.11
	T3	1.05 (0.47, 1.21)	0.66	1.08 (0.67, 1.76)	0.74	0.65 (0.37, 1.15)	0.14	0.94 (0.51, 1.74)	0.86

^1^ Tertile ranges for beta-cryptoxanthin plasma concentrations (mmol/L): T1: <0.218, T2: 0.218–0.415, T3 > 0.415. ^2^ Model 1 contains unadjusted odds ratios, 95% confidence intervals and *p*-values. ^3^ Model 2 contains odds ratios, 95% confidence intervals and *p*-values adjusted for age, ethnicity, log-transformed BMI, plasma triglycerides levels, physical activity level and use of analgesics.

**Table 3:** Unadjusted and adjusted Odds Ratios and 95% CIs for the association between tertiles of plasma beta-carotene and premenstrual symptoms.

Symptom	Tertiles ^1^	Mild vs. None				Moderate/Severe vs. None			
		OR (95% CI)^2^	*p* ^2^	OR (95% CI) ^3^	*p* ^3^	OR (95% CI) ^2^	*p* ^2^	OR (95% CI) ^3^	*p* ^3^
Acne/skin blemish	T1	1	1	1	1	1	1	1	1
	T2	0.96 (0.61, 1.53)	0.88	0.93 (0.57, 1.50)	0.76	1.26 (0.72, 2.18)	0.42	1.29 (0.72, 2.31)	0.38
	T3	1.25 (0.80, 1.98)	0.32	1.16 (0.72, 1.89)	0.53	1.29 (0.74, 2.28)	0.37	1.37 (0.75, 2.51)	0.3
Desire to be alone	T1	1	1	1	1	1	1	1	1
	T2	1.19 (0.73, 1.93)	0.47	1.17 (0.71, 1.94)	0.52	0.84 (0.46, 1.55)	0.58	1.00 (0.53, 1.91)	0.99
	T3	0.68 (0.40, 1.13)	0.14	0.64 (0.37, 1.09)	0.1	0.66 (0.35, 1.22)	0.18	0.87 (0.45, 1.69)	0.68
Anxiety/tension/nervousness	T1	1	1	1	1	1	1	1	1
	T2	0.98 (0.61, 1.58)	0.93	0.91 (0.55, 1.50)	0.71	0.66 (0.33, 1.33)	0.25	0.63 (0.31, 1.29)	0.21
	T3	0.86 (053, 1.41)	0.56	0.78 (0.47, 1.34)	0.39	1.16 (0.63, 2.15)	0.63	1.12 (0.58, 2.14)	0.74
Increased appetite/food cravings	T1	1	1	1	1	1	1	1	1
	T2	1.09 (0.66, 1.80)	0.72	1.26 (0.75, 2.12)	0.37	1.28 (0.78, 2.09)	0.32	1.39 (0.74, 2.05)	0.2
	T3	1.19 (0.72, 1.98)	0.48	1.38 (0.82, 2.36)	0.23	1.54 (0.94, 2.51)	0.08	1.70 (1.01, 2.84)	0.04
Bloating/swelling/breast tenderness	T1	1	1	1	1	1	1	1	1
	T2	0.82 (0.50, 1.36)	0.45	0.81 (0.48, 1.37)	0.44	0.77 (0.46, 1.31)	0.34	0.85 (0.50, 1.49)	0.6
	T3	0.82 (0.49, 1.36)	0.44	0.99 (0.47, 1.38)	0.44	0.87 (0.52, 1.48)	0.62	0.99 (0.57, 1.74)	0.99
Clumsiness	T1	1	1	1	1	1	1	1	1
	T2	0.86 (0.46, 1.59)	0.62	0.84 (0.44, 1.60)	0.6	0.69 (0.22, 2.24)	0.54	0.84 (0.24, 2.91)	0.78
	T3	0.83 (0.45, 1.55)	0.57	0.79 (0.40, 1.52)	0.48	1.28 (0.46, 3.52)	0.64	1.70 (0.55, 5.24)	0.35
Confusion/difficulty concentrating/forgetfulness	T1	1	1	1	1	1	1	1	1
	T2	1.16 (0.65, 2.05)	0.7	1.14 (0.62, 2.08)	0.67	0.43 (0.18, 1.03)	0.05	0.42 (0.17, 1.02)	0.05
	T3	1.19 (0.67, 2.11)	0.55	1.09 (0.59, 2.03)	0.76	0.97 (0.48, 1.96)	0.94	0.96 (0.50, 2.03)	0.91
Cramps	T1	1	1	1	1	1	1	1	1
	T2	1.09 (0.62, 1.91)	0.76	1.29 (0.72, 2.32)	0.39	0.63 (0.45, 1.33)	0.08	0.39 (0.44, 1.38)	0.39
	T3	0.88 (0.50, 1.54)	0.66	1.00 (0.55, 1.81)	0.99	0.58 (0.34, 0.98)	0.04	0.20 (0.39, 1.22)	0.2
Depression	T1	1	1	1	1	1	1	1	1
	T2	1.12 (0.67, 1.88)	0.66	1.13 (0.65, 1.94)	0.66	0.81 (0.40, 1.63)	0.55	0.81 (0.39, 1.67)	0.57
	T3	0.64 (0.36, 1.14)	0.13	0.66 (0.36, 1.21)	0.18	0.98 (0.51, 1.90)	0.96	1.00 (0.50, 2.03)	0.98
Fatigue	T1	1	1	1	1	1	1	1	1
	T2	1.09 (0.68, 1.76)	0.71	1.07 (0.66, 1.76)	0.77	1.37 (0.82, 2.31)	0.23	1.39 (0.81, 2.39)	0.24
	T3	1.16 (0.72, 1.85)	0.54	1.15 (0.70, 1.89)	0.57	1.21 (0.72, 2.06)	0.47	1.18 (0.67, 2.07)	0.56
Headaches	T1	1	1	1	1	1	1	1	1
	T2	1.27 (0.73, 2.20)	0.4	1.58 (0.87, 2.85)	0.13	0.33 (0.15, 0.73)	0.006	0.29 (0.13, 0.68)	0.004
	T3	0.94 (0.52, 1.68)	0.82	1.22 (0.65, 2.30)	0.54	0.57 (0.29, 1.12)	0.1	0.58 (0.28, 1.21)	0.15
Insomnia	T1	1	1	1	1	1	1	1	1
	T2	1.15 (0.58, 2.29)	0.68	1.23 (0.62, 2.61)	0.51	0.22 (0.04, 1.03)	0.053	0.22 (0.04, 1.04)	0.056
	T3	0.86 (0.41, 1.77)	0.86	0.94 (0.43, 2.04)	0.87	0.54 (0.18, 1.64)	0.27	0.58 (0.18, 1.92)	0.37
Mood swings/crying easily/irritability/angry outbursts	T1	1	1	1	1	1	1	1	1
	T2	1.06 (0.63, 1.77)	0.83	0.94 (0.55, 1.60)	0.81	1.18 (0.69, 2.00)	0.55	1.21 (0.70, 2.10)	0.5
	T3	0.75 (0.45, 1.24)	0.26	0.62 (0.36, 1.07)	0.08	0.97 (0.58, 1.62)	0.9	1.04 (0.60, 1.80)	0.9
Nausea	T1	1	1	1	1	1	1	1	1
	T2	0.76 (0.39, 1.49)	0.43	0.81 (0.40, 1.63)	0.55	1.23 (0.47, 3.21)	0.66	1.53 (0.55, 4.25)	0.41
	T3	0.85 (0.44, 1.63)	0.62	0.90 (0.45, 1.81)	0.77	0.86 (0.30, 2.42)	0.77	1.14 (0.37, 3.50)	0.82
Sexual desire/activity change	T1	1	1	1	1	1	1	1	1
	T2	0.81 (0.51, 1.28)	0.36	0.80 (0.49, 1.29)	0.34	0.68 (0.39, 1.21)	0.19	0.80 (0.44, 1.46)	0.46
	T3	0.81 (0.51, 1.29)	0.38	0.83 (0.51, 1.34)	0.44	0.63 (0.35, 1.13)	0.12	0.80 (0.43, 1.50)	0.5

^1^ Tertile ranges for beta-carotene plasma concentrations (mmol/L): T1: <0.503, T2: 0.503–0.933, T3 > 0.933. ^2^ Model 1 contains unadjusted odds ratios, 95% confidence intervals and *p*-values. ^3^ Model 2 contains odds ratios, 95% confidence intervals and *p*-values adjusted for age, ethnicity, log-transformed BMI, plasma triglycerides levels, physical activity level and use of analgesics.

**Table 4:** Unadjusted and adjusted Odds Ratios and 95% CIs for the association between tertiles of plasma alpha-carotene and premenstrual symptoms.

Symptom	Tertiles ^1^	Mild vs. None				Moderate/Severe vs. None			
		OR (95% CI) ^2^	*p* ^2^	OR (95% CI) ^3^	*p* ^3^	OR (95% CI) ^2^	*p* ^2^	OR (95% CI) ^3^	*p* ^3^
Acne/skin blemish	T1	1	1	1	1	1	1	1	1
	T2	1.33 (0.84, 2.10)	0.22	1.35 (0.85, 2.16)	0.2	1.04 (0.60, 1.82)	0.89	1.11 (0.63, 1.97)	0.71
	T3	1.00 (0.63, 1.58)	0.99	0.89 (0.56, 1.43)	0.64	1.00 (0.58, 1.73)	0.99	0.95 (0.54, 1.66)	0.85
Desire to be alone	T1	1	1	1	1	1	1	1	1
	T2	1.18 (0.73, 1.92)	0.49	1.16 (0.71, 1.90)	0.55	0.53 (0.29, 0.99)	0.047	0.54 (0.29, 1.02)	0.058
	T3	0.72 (0.43, 1.21)	0.22	0.73 (0.43, 1.23)	0.23	0.48 (0.26, 0.90)	0.02	0.55 (0.29, 1.04)	0.06
Anxiety/tension/nervousness	T1	1	1	1	1	1	1	1	1
	T2	1.11 (0.70, 1.78)	0.06	1.09 (0.68, 1.75)	0.72	0.61 (0.32, 1.19)	0.15	0.60 (0.31, 1.18)	0.14
	T3	0.60 (0.36, 1.01)	0.054	0.58 (0.35, 0.98)	0.043	0.72 (0.39, 1.33)	0.29	0.68 (0.36, 1.29)	0.24
Increased appetite/food cravings	T1	1	1	1	1	1	1	1	1
	T2	1.12 (0.69, 1.85)	0.64	1.22 (0.74, 2.01)	0.44	1.16 (0.71, 1.89)	0.54	1.21 (0.74, 1.98)	0.45
	T3	1.07 (0.64, 1.77)	0.8	1.10 (0.66, 1.85)	0.71	1.34 (0.82, 2.17)	0.24	1.35 (0.82, 2.22)	0.23
Bloating/swelling/breast tenderness	T1	1	1	1	1	1	1	1	1
	T2	0.87 (0.52, 1.44)	0.58	0.86 (0.52, 1.44)	0.57	0.74 (0.44, 1.25)	0.26	0.79 (0.46, 1.35)	0.39
	T3	0.79 (0.48, 1.32)	0.37	0.78 (0.47, 1.31)	0.35	0.71 (0.42, 1.21)	0.21	0.73 (0.43, 1.25)	0.25
Clumsiness	T1	1	1	1	1	1	1	1	1
	T2	1.25 (0.67, 2.31)	0.48	1.21 (0.65, 2.26)	0.56	0.56 (1.83, 1.71)	0.31	0.62 (0.19, 1.94)	0.41
	T3	1.01 (0.53, 1.94)	0.95	1.04 (0.54, 2.01)	0.91	0.79 (0.29, 2.18)	0.65	0.87 (0.30, 2.47)	0.79
Confusion/difficulty concentrating/forgetfulness	T1	1	1	1	1	1	1	1	1
	T2	1.49 (0.85, 2.62)	0.16	1.43 (0.81, 2.55)	0.22	0.69 (0.32, 1.49)	0.34	0.68 (0.31, 1.49)	0.34
	T3	1.05 (0.58, 1.90)	0.88	1.06 (0.58, 1.96)	0.85	0.73 (0.34, 1.54)	0.4	0.74 (0.34, 1.60)	0.45
Cramps	T1	1	1	1	1	1	1	1	1
	T2	1.01 (0.57, 1.78)	0.96	1.08 (0.61, 1.93)	0.78	0.79 (0.40, 1.47)	0.4	0.90 (0.52, 1.56)	0.71
	T3	0.84 (0.48, 1.46)	0.53	0.86 (0.49, 1.51)	0.6	0.57 (0.33, 0.96)	0.034	0.59 (0.34, 1.03)	0.06
Depression	T1	1	1	1	1	1	1	1	1
	T2	0.92 (0.55, 1.54)	0.76	0.93 (0.55, 1.57)	0.78	0.69 (0.35, 1.37)	0.29	0.67 (0.34, 1.36)	0.27
	T3	0.48 (0.27, 0.86)	0.012	0.46 (0.26, 0.84)	0.011	0.76 (0.39, 1.48)	0.43	0.82 (0.42, 1.60)	0.55
Fatigue	T1	1	1	1	1	1	1	1	1
	T2	1.10 (0.69, 1.77)	0.67	1.07 0.67, 1.73)	0.76	1.55 (0.92, 2.64)	0.1	1.52 (0.89, 2.59)	0.13
	T3	0.93 (0.58, 1.50)	0.78	0.95 (0.59, 1.54)	0.85	1.34 (0.79, 2.28)	0.28	1.40 (0.81, 2.41)	0.23
Headaches	T1	1	1	1	1	1	1	1	1
	T2	1.09 (0.63, 1.91)	0.74	1.13 (0.64, 2.00)	0.67	0.59 (0.29, 1.19)	0.14	0.60 (0.29, 1.25)	0.17
	T3	0.93 (0.53, 1.65)	0.8	1.09 (0.61, 1.98)	0.75	0.59 (0.29, 1.19)	0.14	0.61 (0.29, 1.27)	0.19
Insomnia	T1	1	1	1	1	1	1	1	1
	T2	0.93 (0.47, 1.84)	0.83	0.93 (0.46, 1.88)	0.85	0.70 (0.22, 2.25)	0.55	0.77 (0.23, 2.58)	0.68
	T3	0.78 (0.38, 1.59)	0.49	0.82 (0.40, 1.72)	0.61	0.56 (0.16, 1.97)	0.27	0.61 (0.71, 2.21)	0.46
Mood swings/crying easily/irritability/angry outbursts	T1	1	1	1	1	1	1	1	1
	T2	1.30 (0.77, 2.19)	0.32	1.25 (0.74, 2.12)	0.41	1.35 (0.80, 2.29)	0.26	1.37 (0.80, 2.34)	0.25
	T3	0.86 (0.52, 1.44)	0.57	0.79 (0.47, 1.33)	0.37	0.92 (0.55, 1.53)	0.74	0.94 (0.56, 1.59)	0.82
Nausea	T1	1	1	1	1	1	1	1	1
	T2	0.35 (0.17, 0.73)	0.005	0.38 (0.18, 0.80)	0.01	0.90 (0.35, 2.32)	0.82	1.11 (0.41, 3.02)	0.83
	T3	0.65 (0.35, 1.22)	0.18	0.64 (0.33, 1.23)	0.18	0.75 (0.27, 2.06)	0.57	0.82 (0.28, 2.36)	0.72
Sexual desire/activity change	T1	1	1	1	1	1	1	1	1
	T2	1.77 (1.11, 2.81)	0.015	1.80 (1.13, 2.88)	0.01	0.56 (0.31, 1.03)	0.06	0.64 (0.34, 1.19)	0.16
	T3	1.07 (0.66, 1.74)	0.78	1.06 (0.65, 1.73)	0.81	0.75 (0.43, 1.29)	0.3	0.81 (0.46, 1.44)	0.47

^1^ Tertile ranges for alpha-carotene plasma concentrations (mmol/L): T1: <0.118, T2: 0.118–0.241, T3 > 0.241. ^2^ Model 1 contains unadjusted odds ratios, 95% confidence intervals and *p*-values. ^3^ Model 2 contains odds ratios, 95% confidence intervals and p-values adjusted for age, ethnicity, log-transformed BMI, plasma triglycerides levels, physical activity level and use of analgesics.

**Table 5:** Unadjusted and adjusted Odds Ratios and 95% CIs for the association between tertiles of plasma retinol and premenstrual symptoms.

Symptom	Tertiles ^1^	Mild vs. None				Moderate/Severe vs. None			
		OR (95% CI) ^2^	*p* ^2^	OR (95% CI) ^3^	*p* ^3^	OR (95% CI) ^2^	*p* ^2^	OR (95% CI) ^3^	*p* ^3^
Acne/skin blemish	T1	1	1	1	1	1	1	1	1
	T2	1.30 (0.83, 2.04)	0.25	1.37 (0.86, 2.19)	0.18	0.88 (0.49, 1.59)	0.68	0.81 (0.44, 1.48)	0.49
	T3	0.84 (0.53, 1.34)	0.47	0.83 (0.51, 1.36)	0.47	1.35 (0.79, 2.31)	0.27	1.20 (0.69, 2.12)	0.51
Desire to be alone	T1	1	1	1	1	1	1	1	1
	T2	1.44 (0.88, 2.35)	0.14	1.48 (0.90, 2.45)	0.12	1.76 (0.92, 3.38)	0.08	1.63 (0.83, 3.18)	0.15
	T3	1.08 (0.66, 1.80)	0.75	1.20 (0.71, 2.04)	0.49	1.63 (0.85, 3.11)	0.14	1.57 (0.80, 3.10)	0.19
Anxiety/tension/nervousness	T1	1	1	1	1	1	1	1	1
	T2	1.11 (0.70, 1.78)	0.06	0.95 (0.59, 1.55)	0.85	0.61 (0.32, 1.19)	0.15	1.53 (0.79, 2.99)	0.21
	T3	0.60 (0.36, 1.01)	0.054	1.04 (0.64, 1.68)	0.88	0.72 (0.39, 1.33)	0.29	1.50 (0.76, 2.93)	0.24
Increased appetite/food cravings	T1	1	1	1	1	1	1	1	1
	T2	1.03 (0.62, 1.70)	0.91	0.98 (0.59, 1.64)	0.95	1.19 (0.73, 1.93)	0.49	1.19 (0.73, 1.96)	0.48
	T3	1.29 (0.78, 2.13)	0.32	1.19 (0.70, 2.01)	0.52	1.44 (0.88, 2.36)	0.14	1.46 (0.88, 2.44)	0.14
Bloating/swelling/breast tenderness	T1	1	1	1	1	1	1	1	1
	T2	1.25 (0.76, 2.07)	0.38	1.27 (0.76, 2.12)	0.35	1.27 (0.74, 2.16)	0.37	1.17 (0.68, 2.01)	0.57
	T3	0.97 (0.59, 1.60)	0.91	0.96 (0.57, 1.61)	0.86	1.27 (0.76, 2.12)	0.37	1.17 (0.68, 2.00)	0.57
Clumsiness	T1	1	1	1	1	1	1	1	1
	T2	0.75 (0.40, 1.42)	0.38	0.72 (0.37, 1.38)	0.32	1.73 (0.50, 6.02)	0.39	1.44 (0.40, 5.19)	0.58
	T3	0.99 (0.55, 1.83)	0.99	0.96 (0.51, 1.81)	0.91	2.60 (0.80, 8.47)	0.11	2.21 (0.65, 7.56)	0.2
Confusion/difficulty concentrating/forgetfulness	T1	1	1	1	1	1	1	1	1
	T2	0.84 (0.48, 1.45)	0.54	0.83 (0.47, 1.46)	0.52	0.68 (0.29, 1.58)	0.37	0.58 (0.24, 1.39)	0.22
	T3	0.75 (0.42, 1.32)	0.31	0.76 (0.41, 1.38)	0.36	1.34 (0.65, 2.77)	0.43	1.26 (0.59, 2.72)	0.55
Cramps	T1	1	1	1	1	1	1	1	1
	T2	0.92 (0.53, 1.61)	0.78	0.84 (0.47, 1.49)	0.55	0.94 (0.55, 1.60)	0.82	0.82 (0.47, 1.43)	0.48
	T3	0.83 (0.48, 1.44)	0.51	0.71 (0.40, 1.27)	0.25	0.74 (0.44, 1.25)	0.26	0.62 (0.35, 1.09)	0.09
Depression	T1	1	1	1	1	1	1	1	1
	T2	1.82 (1.05, 3.14)	0.03	1.67 (0.95, 2.93)	0.07	1.37 (0.38, 2.78)	0.38	1.37 0.66, 2.81)	0.4
	T3	1.30 (0.73, 2.31)	0.37	1.08 (0.59, 1.97)	0.8	1.50 (0.76, 2.98)	0.25	1.64 (0.80, 3.36)	0.18
Fatigue	T1	1	1	1	1	1	1	1	1
	T2	0.79 (0.50, 1.27)	0.33	0.82 (0.50, 1.32)	0.41	0.94 (0.56, 1.59)	0.81	0.95 (0.55, 1.63)	0.85
	T3	0.72 (0.45, 1.16)	0.17	0.77 (0.47, 1.26)	0.29	0.96 (0.57, 1.62)	0.88	1.08 (0.63, 1.86)	0.78
Headaches	T1	1	1	1	1	1	1	1	1
	T2	1.05 (0.61, 1.83)	0.85	0.91 (0.52, 1.61)	0.75	1.08 (0.53, 2.24)	0.82	0.84 (0.39, 1.78)	0.64
	T3	0.86 (0.49, 1.52)	0.61	0.82 (0.45, 1.51)	0.53	1.12 (0.55, 2.28)	0.76	0.88 (0.41, 1.87)	0.74
Insomnia	T1	1	1	1	1	1	1	1	1
	T2	0.95 (0.48, 1.88)	0.89	0.91 (0.45, 1.85)	0.8	1.20 (0.36, 4.04)	0.76	1.10 (0.32, 3.85)	0.87
	T3	0.77 (0.38, 1.58)	0.48	0.86 (0.41, 1.83)	0.7	0.98 (0.28, 3.46)	0.98	1.12 (0.30, 4.21)	0.86
Mood swings/crying easily/irritability/angry outbursts	T1	1	1	1	1	1	1	1	1
	T2	0.72 (0.43, 1.20)	0.2	0.79 (0.47, 1.34)	0.38	1.21 (0.71, 2.04)	0.48	1.18 (0.70, 2.02)	0.53
	T3	0.91 (0.54, 1.52)	0.71	1.06 (0.62, 1.83)	0.82	1.41 (0.83, 2.39)	0.21	1.47 (0.84, 2.56)	0.18
Nausea	T1	1	1	1	1	1	1	1	1
	T2	1.04 (0.54, 2.01)	0.91	0.92 (0.47, 1.82)	0.82	1.91 (0.69, 5.28)	0.21	1.52 (0.52, 4.41)	0.44
	T3	0.91 (0.46, 1.78)	0.77	0.78 (0.38, 1.58)	0.49	1.34 (0.45, 3.96)	0.59	1.16 (0.37, 3.62)	0.8
Sexual desire/activity change	T1	1	1	1	1	1	1	1	1
	T2	1.12 (0.70, 1.79)	0.64	1.10 (0.69, 1.79)	0.67	2.35 (1.31, 4.23)	0.004	2.17 (1.17, 4.01)	0.014
	T3	1.40 (0.89, 2.20)	0.14	1.41 (0.88, 2.27)	0.15	2.52 (0.81, 2.83)	0.19	1.41 (0.73, 2.75)	0.31

^1^ Tertile ranges for retinol plasma concentrations (mmol/L): T1: <1.490, T2: 1.490–1.806, T3 > 1.806. ^2^ Model 1 contains unadjusted odds ratios, 95% confidence intervals and *p*-values. ^3^ Model 2 contains odds ratios, 95% confidence intervals and *p*-values adjusted for age, ethnicity, log-transformed BMI, plasma triglycerides levels, physical activity level and use of analgesics.

**Table 6:** Unadjusted and adjusted Odds Ratios and 95% CIs for the association between tertiles of plasma lutein and premenstrual symptoms.

Symptom	Tertiles ^1^	Mild vs. None				Moderate/Severe vs. None			
		OR (95% CI) ^2^	*p* ^2^	OR (95% CI) ^3^	*p* ^3^	OR (95% CI) ^2^	*p* ^2^	OR (95% CI) ^3^	*p* ^3^
Acne/skin blemish	T1	1	1	1	1	1	1	1	1
	T2	0.95 (0.60, 1.50)	0.82	0.95 (0.59, 1.53)	0.84	0.95 (0.55, 1.65)	0.85	1.01 (0.57, 1.79)	0.97
	T3	1.10 (0.70, 1.74)	0.68	1.07 (0.66, 1.73)	0.78	1.05 (0.61, 1.84)	0.85	1.12 (0.63, 2.00)	0.71
Desire to be alone	T1	1	1	1	1	1	1	1	1
	T2	0.98 (0.60, 1.59)	0.93	0.97 (0.59, 1.59)	0.9	0.71 (0.38, 1.34)	0.29	0.78 (0.41, 1.50)	0.15
	T3	0.79 (0.48, 1.30)	0.35	0.77 (0.46, 1.29)	0.31	0.86 (0.47, 1.58)	0.64	1.05 (0.56, 1.97)	0.19
Anxiety/tension/nervousness	T1	1	1	1	1	1	1	1	1
	T2	0.67 (0.41, 1.08)	0.1	0.62 (0.38, 1.02)	0.059	0.81 (0.42, 1.56)	0.52	0.81 (0.42, 1.59)	0.55
	T3	0.83 (0.52, 1.35)	0.46	0.76 (0.46, 1.25)	0.29	1.05 (0.56, 1.99)	0.87	1.02 (0.52, 1.98)	0.96
Increased appetite/food cravings	T1	1	1	1	1	1	1	1	1
	T2	0.85 (0.52, 1.41)	0.53	0.92 (0.55, 1.53)	0.74	0.87 (0.50, 1.20)	0.21	0.76 (0.46, 1.25)	0.28
	T3	0.74 (0.53, 1.45)	0.6	0.99 (0.58, 1.68)	0.97	0.93 (0.57, 1.51)	0.76	0.98 (0.59, 1.62)	0.94
Bloating/swelling/breast tenderness	T1	1	1	1	1	1	1	1	1
	T2	0.95 (0.57, 1.57)	0.84	0.95 (0.57, 1.60)	0.86	0.93 (0.55, 1.57)	0.79	1.04 (0.61, 1.78)	0.9
	T3	0.83 (0.50, 1.38)	0.47	0.83 (0.49, 1.39)	0.48	0.86 (0.51, 1.45)	0.58	0.98 (0.57, 1.70)	0.96
Clumsiness	T1	1	1	1	1	1	1	1	1
	T2	0.62 (0.32, 1.18)	0.15	0.55 (0.28, 1.08)	0.08	0.83 (0.29, 2.33)	0.72	0.82 (0.28, 2.40)	0.71
	T3	0.95 (0.52, 1.72)	0.86	0.87 (0.47, 1.62)	0.67	0.74 (0.25, 2.18)	0.69	0.82 (0.26, 2.54)	0.73
Confusion/difficulty concentrating/forgetfulness	T1	1	1	1	1	1	1	1	1
	T2	0.85 (0.48, 1.52)	0.59	0.74 (0.41, 1.34)	0.32	1.07 (0.47, 2.42)	0.87	1.04 (0.45, 2.41)	0.93
	T3	1.09 (0.63, 1.91)	0.75	0.87 (0.54, 1.72)	0.91	1.59 (0.74, 3.42)	0.24	1.59 (0.71, 3.56)	0.25
Cramps	T1	1	1	1	1	1	1	1	1
	T2	1.16 (0.65, 2.08)	0.61	1.32 0.73, 2.38)	0.36	1.00 (0.55, 1.60)	0.99	1.24 (0.71, 2.20)	0.45
	T3	0.76 (0.44, 2.08)	0.32	0.88 (0.50, 1.54)	0.65	0.48 (0.29, 0.81)	0.006	0.62 (0.35, 1.07)	0.08
Depression	T1	1	1	1	1	1	1	1	1
	T2	0.85 (0.50, 1.46)	0.56	0.83 (0.47, 1.44)	0.5	0.86 (0.43, 1.73)	0.68	0.88 (0.72, 0.43)	0.72
	T3	0.91 (0.53, 1.56)	0.74	0.90 (0.52, 1.58)	0.71	1.10 (0.57, 2.15)	0.77	1.12 (0.56, 2.26)	0.74
Fatigue	T1	1	1	1	1	1	1	1	1
	T2	0.89 (0.55, 1.42)	0.62	0.86 (0.53, 1.40)	0.54	1.15 (0.68, 1.94)	0.6	1.11 (0.65, 1.90)	0.69
	T3	0.98 (0.61, 1.56)	0.92	0.94 (0.58, 1.52)	0.8	1.13 (0.67, 1.91)	0.66	1.08 (0.63, 1.88)	0.77
Headaches	T1	1	1	1	1	1	1	1	1
	T2	0.74 (0.42, 1.31)	0.3	0.83 (0.46, 1.49)	0.53	0.84 (0.41, 1.71)	0.63	0.85 (0.41, 1.79)	0.68
	T3	0.93 (0.54, 1.60)	0.78	1.12 (0.63, 1.99)	0.71	0.93 (0.46, 1.88)	0.84	0.98 (0.47, 2.05)	0.95
Insomnia	T1	1	1	1	1	1	1	1	1
	T2	0.50 (0.24, 1.04)	0.06	0.54 (0.25, 1.14)	0.1	1.93 (0.57, 6.53)	0.29	2.36 (0.67, 8.35)	0.18
	T3	0.72 (0.37, 1.39)	0.32	0.77 (0.38, 1.56)	0.47	0.97 (0.24, 3.94)	0.97	1.23 (0.28, 5.35)	0.78
Mood swings/crying easily/irritability/angry outbursts	T1	1	1	1	1	1	1	1	1
	T2	0.53 (0.32, 0.88)	0.015	0.49 (0.28, 0.83)	0.008	0.85 (0.50, 1.43)	0.53	0.89 (0.52, 1.53)	0.68
	T3	0.74 (0.44, 1.25)	0.27	0.66 (0.38, 1.13)	0.13	1.02 (0.60, 1.74)	0.94	1.08 (0.62, 1.89)	0.78
Nausea	T1	1	1	1	1	1	1	1	1
	T2	0.87 (0.45, 1.69)	0.69	0.94 (0.48, 1.85)	0.85	0.67 (00.28, 1.61)	0.37	0.79 (0.31, 1.97)	0.61
	T3	0.79 (0.41, 1.55)	0.5	0.86 (0.42, 1.72)	0.66	0.21 (0.06, 0.76)	0.017	0.25 (0.05, 0.93)	0.04
Sexual desire/activity change	T1	1	1	1	1	1	1	1	1
	T2	0.83 (0.52, 1.32)	0.43	0.87 (0.54, 1.39)	0.55	0.94 (0.54, 1.64)	0.82	1.17 (0.65, 2.13)	0.59
	T3	0.90 (0.57, 1.42)	0.64	0.92 (0.58, 1.48)	0.74	0.76 (0.42, 1.36)	0.35	0.99 (0.53, 1.85)	0.98

^1^ Tertile ranges for lutein plasma concentrations (mmol/L): T1: <0.146, T2: 0.146–0.480, T3 > 0.480. ^2^ Model 1 contains unadjusted odds ratios, 95% confidence intervals and *p*-values. ^3^ Model 2 contains odds ratios, 95% confidence intervals and *p*-values adjusted for age, ethnicity, log-transformed BMI, plasma triglycerides levels, physical activity level and use of analgesics.

**Table 7:** Unadjusted and adjusted Odds Ratios and 95% CIs for the association between tertiles of plasma zeaxanthin and premenstrual symptoms.

Symptom	Tertiles ^1^	Mild vs. None				Moderate/Severe vs. None			
		OR (95% CI)^2^	*p* ^2^	OR (95% CI) ^3^	*p* ^3^	OR (95% CI) ^2^	*p* ^2^	OR (95% CI) ^3^	*p* ^3^
Acne/skin blemish	T1	1	1	1	1	1	1	1	1
	T2	1.30 (0.83, 2.05)	0.25	1.32 (0.83, 2.09)	0.24	1.02 (0.58, 1.81)	0.93	1.04 (0.58, 1.85)	0.9
	T3	1.08 (0.69, 1.72)	0.72	1.07 (0.67, 1.71)	0.76	1.30 (0.76, 2.23)	0.34	1.35 (0.78, 2.34)	0.28
Desire to be alone	T1	1	1	1	1	1	1	1	1
	T2	0.78 (0.48, 1.27)	0.31	0.78 (0.48, 1.28)	0.33	1.33 (0.70, 2.52)	0.39	1.36 (0.71, 2.62)	0.35
	T3	0.78 (0.48, 1.26)	0.31	0.77 (0.47, 1.25)	0.29	1.40 (0.74, 2.63)	0.3	1.55 (0.81, 2.95)	0.19
Anxiety/tension/nervousness	T1	1	1	1	1	1	1	1	1
	T2	0.73 (0.45, 1.19)	0.21	0.73 (0.45, 1.20)	0.22	1.13 (0.59, 2.17)	0.72	1.15 (0.59, 2.32)	0.68
	T3	0.98 (0.61, 1.56)	0.92	0.96 (0.59, 1.54)	0.85	1.30 (0.68, 2.48)	0.43	1.30 (0.68, 2.51)	0.43
Increased appetite/food cravings	T1	1	1	1	1	1	1	1	1
	T2	0.99 (0.60, 1.62)	0.96	0.98 (0.60, 1.62)	0.94	1.04 (0.64, 1.71)	0.64	1.05 (0.64, 1.71)	0.85
	T3	0.98 (0.59, 1.61)	0.93	1.02 (0.62, 1.70)	0.93	1.27 (0.78, 2.05)	0.33	1.30 (0.80, 2.11)	0.29
Bloating/swelling/breast tenderness	T1	1	1	1	1	1	1	1	1
	T2	1.00 (0.61, 1.66)	0.97	1.00 (0.61, 1.65)	0.99	1.01 (0.60, 1.70)	0.98	1.02 (0.60, 1.73)	0.94
	T3	1.07 (0.65, 1.77)	0.79	1.07 (0.64, 1.77)	0.8	1.24 (0.74, 2.08)	0.42	1.33 (0.79, 2.25)	0.28
Clumsiness	T1	1	1	1	1	1	1	1	1
	T2	1.53 (0.82, 2.84)	0.18	1.50 (0.80, 2.80)	0.21	0.63 (0.21, 1.92)	0.41	0.61 (0.19, 1.92)	0.4
	T3	1.11 (0.58, 2.13)	0.75	1.06 (0.55, 2.04)	0.86	0.82 (0.30, 2.26)	0.71	0.89 (0.32, 2.51)	0.83
Confusion/difficulty concentrating/forgetfulness	T1	1	1	1	1	1	1	1	1
	T2	0.95 (0.54, 1.68)	0.87	0.93 (0.52, 1.65)	0.8	1.18 (0.54, 2.60)	0.68	1.20 (0.54, 2.68)	0.65
	T3	1.01 (0.58, 1.76)	0.97	0.94 (0.54, 1.66)	0.84	1.33 (0.62, 2.86)	0.47	1.34 (0.62, 2.93)	0.46
Cramps	T1	1	1	1	1	1	1	1	1
	T2	0.65 (0.38, 1.14)	0.13	0.63 (0.36, 1.11)	0.11	0.59 (0.35, 1.01)	0.054	0.57 (0.33, 0.99)	0.044
	T3	0.78 (0.45, 1.36)	0.38	0.81 (0.46, 1.42)	0.46	0.73 (0.43, 1.24)	0.24	0.79 (0.45, 1.37)	0.4
Depression	T1	1	1	1	1	1	1	1	1
	T2	1.39 (0.82, 2.36)	0.23	1.36 (0.79, 2.32)	0.27	1.42 (0.70, 2.89)	0.33	1.42 (0.70, 2.90)	0.33
	T3	0.99 (0.57, 1.73)	0.99	0.98 (0.56, 1.72)	0.95	1.51 (0.76, 3.00)	0.24	1.53 (0.77, 3.05)	0.23
Fatigue	T1	1	1	1	1	1	1	1	1
	T2	1.15 (0.72, 1.84)	0.57	1.16 (0.72, 1.87)	0.54	1.15 (0.66, 1.88)	0.68	1.14 (0.67, 1.92)	0.63
	T3	1.13 (0.70, 1.80)	0.62	1.12 (0.70, 1.80)	0.64	1.13 (0.67, 1.88)	0.65	1.11 (0.66, 1.87)	0.7
Headaches	T1	1	1	1	1	1	1	1	1
	T2	0.72 (0.41, 1.27)	0.25	0.71 (0.40, 1.27)	0.24	1.10 (0.55, 2.19)	0.78	1.06 (0.53, 2.15)	0.86
	T3	0.90 (0.53, 1.54)	0.7	0.93 (0.54, 1.61)	0.79	0.79 (0.38, 1.65)	0.53	0.79 (0.37, 1.69)	0.55
Insomnia	T1	1	1	1	1	1	1	1	1
	T2	1.07 (0.55, 2.10)	0.84	1.09 (0.55, 2.16)	0.8	0.40 (0.10, 1.54)	0.18	0.41 (0.10, 1.58)	0.19
	T3	0.75 (0.36, 1.54)	0.43	0.74 (0.35, 1.54)	0.52	0.63 (0.20, 1.97)	0.43	0.65 (0.21, 2.08)	0.47
Mood swings/crying easily/irritability/angry outbursts	T1	1	1	1	1	1	1	1	1
	T2	0.74 (0.44, 1.23)	0.25	0.74 (0.44, 1.25)	0.26	0.91 (0.54, 1.54)	0.73	0.92 (0.54, 1.55)	0.76
	T3	0.81 (0.49, 1.36	0.43	0.77 (0.46, 1.30)	0.34	0.96 (0.57, 1.62)	0.88	0.98 (0.58, 1.65)	0.93
Nausea	T1	1	1	1	1	1	1	1	1
	T2	0.83 (0.42, 1.64)	0.59	0.84 (0.42, 1.70)	0.63	1.40 (0.51, 3.84)	0.52	1.47 (0.52, 4.17)	0.47
	T3	1.08 (0.57, 2.05)	0.82	1.18 (0.61, 2.28)	0.62	1.39 (0.50, 3.81)	0.53	1.63 (0.57, 4.67)	0.36
Sexual desire/activity change	T1	1	1	1	1	1	1	1	1
	T2	1.14 (0.72, 1.81)	0.57	1.15 (0.72, 1.82)	0.56	1.36 (0.77, 2.40)	0.29	1.45 (0.80, 2.62)	0.22
	T3	1.01 (0.64, 1.59)	0.96	1.02 (0.65, 1.61)	0.93	1.03 (0.57, 1.84)	0.93	1.20 (0.66, 2.19)	0.56

^1^ Tertile ranges for zeaxanthin plasma concentrations (mmol/L): T1: <0.078, T2: 0.078–0.127, T3 > 0.127. ^2^ Model 1 contains unadjusted odds ratios, 95% confidence intervals and *p*-values. ^3^ Model 2 contains odds ratios, 95% confidence intervals and *p*-values adjusted for age, ethnicity, log-transformed BMI, plasma triglycerides levels, physical activity level and use of analgesics.

**Table 8:** Unadjusted and adjusted Odds Ratios and 95% CIs for the association between tertiles of plasma trans-lycopene and premenstrual symptoms.

Symptom	Tertiles ^1^	Mild vs. None				Moderate/Severe vs. None			
		OR (95% CI) ^2^	*p* ^2^	OR (95% CI) ^3^	*p* ^3^	OR (95% CI) ^2^	*p* ^2^	OR (95% CI) ^3^	*p* ^3^
Acne/skin blemish	T1	1	1	1	1	1	1	1	1
	T2	1.34 (0.84, 2.13)	0.22	1.32 (0.82, 2.12)	0.25	1.79 (1.03, 3.09)	0.038	1.73 (1.00, 3.03)	0.053
	T3	1.03 (0.66m 1.62)	0.9	1.07 (0.67, 1.72)	0.77	0.91 (0.51, 1.62)	0.75	0.94 (0.52, 1.70)	0.84
Desire to be alone	T1	1	1	1	1	1	1	1	1
	T2	0.77 (0.47, 1.26)	0.3	0.78 (0.47, 1.29)	0.33	1.12 (0.60, 2.09)	0.39	1.12 (0.59, 2.11)	0.73
	T3	0.94 (0.58, 1.52)	0.79	0.96 (0.58, 1.57)	0.86	1.09 (0.58, 2.06)	0.3	1.03 (0.54, 1.98)	0.93
Anxiety/tension/nervousness	T1	1	1	1	1	1	1	1	1
	T2	1.04 (0.64, 1.69)	0.86	1.10 (0.67, 1.79)	0.71	0.95 (0.65, 1.69)	0.88	0.95 (0.49, 1.85)	0.88
	T3	1.01 (0.62, 1.65)	0.96	1.13 (0.68, 1.86)	0.64	1.23 (0.65, 2.32)	0.52	1.34 (0.70, 2.59)	0.38
Increased appetite/food cravings	T1	1	1	1	1	1	1	1	1
	T2	1.27 (0.77, 2.10)	0.36	1.26 (0.66, 1.88)	0.38	1.07 (0.66, 1.74)	0.78	1.08 (0.66, 1.77)	0.75
	T3	1.18 (0.71, 1.96)	0.52	1.11 (0.85, 1.02)	0.68	1.05 (0.65, 1.71)	0.83	1.07 (0.65, 1.75)	0.8
Bloating/swelling/breast tenderness	T1	1	1	1	1	1	1	1	1
	T2	1.24 (0.76, 2.04)	0.4	1.20 (0.73, 1.99)	0.47	1.56 (0.92, 2.63)	0.09	1.49 (0.88, 2.54)	0.14
	T3	1.33 (0.80, 2.20)	0.27	1.31 (0.78, 2.19)	0.31	1.77 (1.04, 3.00)	0.033	1.74 (1.01, 2.99)	0.045
Clumsiness	T1	1	1	1	1	1	1	1	1
	T2	0.66 (0.35, 1.22)	0.18	0.68 (0.36, 1.26)	0.22	0.51 (0.17, 1.56)	0.24	0.52 (0.17, 1.62)	0.26
	T3	0.67 (0.36, 1.25)	0.21	0.73 (0.38, 1.37)	0.32	0.73 (0.27, 2.02)	0.55	0.87 (0.30, 2.47)	0.79
Confusion/difficulty concentrating/forgetfulness	T1	1	1	1	1	1	1	1	1
	T2	1.09 (0.62, 1.92)	0.76	1.20 (0.68, 2.14)	0.53	0.77 (0.37, 1.60)	0.48	0.77 (0.37, 1.63)	0.5
	T3	0.96 (0.54, 1.70)	0.88	1.11 (0.61, 2.01)	0.73	0.59 (0.27, 1.28)	0.18	0.62 (0.28, 1.40)	0.25
Cramps	T1	1	1	1	1	1	1	1	1
	T2	0.76 (0.44, 1.29)	0.31	0.70 (0.41, 1.21)	0.2	0.99 (0.59, 1.66)	0.96	0.92 (0.53, 1.58)	0.75
	T3	0.98 (0.56, 1.72)	0.96	0.85 (0.48, 1.52)	0.59	0.150 (0.87, 2.56)	0.14	1.21 (0.69, 2.13)	0.51
Depression	T1	1	1	1	1	1	1	1	1
	T2	1.42 (0.84, 2.39)	0.19	1.37 (0.80, 2.34)	0.25	0.81 (0.39, 1.70)	0.59	0.80 (0.28, 1.69)	0.57
	T3	0.82 (0.46, 1.46)	0.5	0.84 (0.46, 1.52)	0.56	1.41 (0.74, 2.70)	0.3	1.44 (0.73, 2.82)	0.29
Fatigue	T1	1	1	1	1	1	1	1	1
	T2	1.24 (0.77, 2.00)	0.38	1.27 (0.78, 2.06)	0.33	0.85 (0.51, 1.43)	0.55	0.89 (0.53, 1.51)	0.68
	T3	1.23 (0.76, 2.00)	0.4	1.23 (0.75, 2.02)	0.41	0.92 (0.55, 1.54)	0.75	0.98 (0.58, 1.66)	0.94
Headaches	T1	1	1	1	1	1	1	1	1
	T2	0.87 (0.49, 1.54)	0.63	0.87 (0.48, 1.56)	0.64	1.31 (0.66, 2.62)	0.44	1.26 (0.62, 2.56)	0.53
	T3	1.16 (0.67, 2.00)	0.6	1.12 (0.63, 1.97)	0.7	0.89 (0.42, 1.90)	0.77	0.86 (0.39, 1.87)	0.7
Insomnia	T1	1	1	1	1	1	1	1	1
	T2	0.93 (0.46, 1.87)	0.85	0.97 (0.48, 1.98)	0.94	0.99 (0.24, 4.02)	0.99	1.00 (0.24, 4.09)	0.99
	T3	0.97 (0.48, 1.95)	0.93	1.06 (0.51, 2.18)	0.88	2.05 (0.61, 6.95)	0.25	2.03 (0.58, 7.16)	0.27
Mood swings/crying easily/irritability/angry outbursts	T1	1	1	1	1	1	1	1	1
	T2	1.08 (0.65, 1.80)	0.76	1.10 (0.66, 1.86)	0.71	1.32 (0.78, 2.25)	0.3	1.31 (0.77, 2.23)	0.32
	T3	0.88 (0.53, 1.47)	0.64	0.92 (0.54, 1.56)	0.75	1.26 (0.75, 2.12)	0.39	1.27 (0.74, 2.16)	0.39
Nausea	T1	1	1	1	1	1	1	1	1
	T2	1.43 (0.71, 2.87)	0.32	1.40 (0.69, 2.84)	0.36	0.82 (0.31, 2.12)	0.68	0.77 (0.29, 2.07)	0.61
	T3	1.51 (0.76, 3.03)	0.24	1.39 (0.68, 2.83)	0.36	0.72 (0.27, 1.95)	0.52	0.63 (0.22, 1.78)	0.39
Sexual desire/activity change	T1	1	1	1	1	1	1	1	1
	T2	1.18 (0.75, 1.86)	0.48	1.15 (0.72, 1.82)	0.56	1.54 (0.85, 2.80)	0.16	1.47 (0.80, 2.73)	0.22
	T3	1.15 (0.72, 1.82)	0.56	1.18 (0.73, 1.90)	0.49	1.72 (0.95, 3.10)	0.07	1.54 (0.83, 2.87)	0.56

^1^ Tertile ranges for trans-lycopene plasma concentrations (mmol/L): T1: <0.472, T2: 0.472–0.836, T3 > 0.836. ^2^ Model 1 contains unadjusted odds ratios, 95% confidence intervals and *p*-values. ^3^ Model 2 contains odds ratios, 95% confidence intervals and *p*-values adjusted for age, ethnicity, log-transformed BMI, plasma triglycerides levels, physical activity level and use of analgesics.

## Data Availability

The data presented in this study are available on request from the corresponding author.
